# Social deprivation modifies the association between incident foot ulceration and mortality in type 1 and type 2 diabetes: a longitudinal study of a primary-care cohort

**DOI:** 10.1007/s00125-017-4522-x

**Published:** 2017-12-21

**Authors:** Simon G. Anderson, Haika Shoo, Sushant Saluja, Christian D. Anderson, Adnan Khan, Mark Livingston, Edward B. Jude, Mark Lunt, George Dunn, Adrian H. Heald

**Affiliations:** 1 Division of Cardiovascular Sciences, Faculty of Biology, Medicine, and Health, Core Technology Facility, The University of Manchester, Manchester, UK; 2 Diabetes and Endocrine Department, East Cheshire NHS Trust, Macclesfield, UK; 3 School of Medicine, University of Liverpool, Liverpool, UK; 4 Department of Endocrinology and Diabetes, Leighton Hospital, Crewe, UK; 5 Department of Blood Sciences, Walsall Manor Hospital, Walsall, UK; 6 Department of Diabetes and Endocrinology, Tameside Hospital NHS Foundation Trust, Ashton-under-Lyme, UK; 7 Arthritis Research UK Centre for Epidemiology, Centre for Musculoskeletal Research, School of Biological Sciences and Manchester Academic Health Science Centre, University of Manchester, Manchester, UK; 8 Department of Podiatry, East Cheshire NHS Trust, Macclesfield, UK; 9 Salford Royal NHS Foundation Trust, Diabetes and Endocrinology, Stott Lane, Salford, UK; 10 School of Medical Sciences, Faculty of Biology, Medicine, and Health, and Manchester Academic Health Science Centre (MAHSC), The University of Manchester, 46 Grafton Street, Manchester, M13 9NT UK

**Keywords:** Deprivation index, Diabetes, Foot ulcer, Mortality

## Abstract

**Aims/hypothesis:**

The aim of this study was to determine whether social deprivation in the presence of diabetes is an independent predictor of developing a foot ulcer and separately of mortality.

**Methods:**

This was a primary-care-based retrospective analysis of 13,955 adults with type 1 (*n* = 1370) or type 2 (*n* = 12,585) diabetes after a median follow-up of 10.5 years. Demographic characteristics, indices of social deprivation and clinical variables were assessed at baseline. The primary outcomes were new foot ulceration (in those without a previous history of foot ulcers) and all-cause mortality. Cox proportional hazard models were used to describe the associations among foot ulceration, social deprivation and mortality.

**Results:**

The mean age of the population was 69.4 (range: 16–89) years. The incidence of foot ulceration was greater in individuals with type 2 (8.6%) compared with type 1 diabetes (4.8%). Occurrence was similar by sex, but increased with age and deprivation index. Individuals in the highest quintile of deprivation were 77% more likely to develop a foot ulcer compared with those in the lowest quintile (OR 1.77 [95% CI 1.45, 2.14], *p* < 0.0001). Overall, 2946 (21.1%) deaths were recorded. Compared with individuals without a foot ulcer, the development of a foot ulcer was associated with a higher age- and sex-adjusted mortality rate (25.9% vs 14.0%), and a 72% (HR 1.72 [95% CI 1.56, 1.90], *p* < 0.001) increased risk of mortality in those with type 2 diabetes. Risk of death increased by 14% per quintile of deprivation in a univariable analysis (HR 1.14 [95% CI 1.10, 1.17]). In multivariable Cox regression analyses, there was a 48% increased risk of mortality in individuals with a foot ulcer (HR 1.48 [95% CI 1.33, 1.66]) independent of the Townsend index score (HR 1.13 [95% CI 1.10, 1.17], per quintile), baseline age, sex, diabetes type, smoking status, hypertension, statin use, β-blocker use, metformin use, HbA_1c_ levels and insulin use.

**Conclusions/interpretation:**

This study confirms the high mortality rate in individuals with diabetes-related foot ulcers. In addition, socioeconomic disadvantage was found to be an independent effect modifier, contributing to an increased burden of mortality in people with diabetes who develop foot ulceration. In light of this, and as diabetes service configurations are orientated for the next 5–10 years, modelling of foot ulceration risk needs to take socioeconomic disadvantage into account.

**Electronic supplementary material:**

The online version of this article (10.1007/s00125-017-4522-x) contains peer-reviewed but unedited supplementary material, which is available to authorised users.

## Introduction

Foot ulceration is the most common complication in diabetes, with a lifetime risk of 25% [1]. The condition portends significant excess morbidity and mortality in individuals with diabetes, who are already facing reduced life expectancy and an unfavourable prognosis [1–5]. Established aetiological risk factors for foot ulceration in diabetes are suboptimally controlled diabetes, peripheral neuropathy, peripheral vascular disease, foot deformity and previous foot ulceration [1, 6].

Diabetic foot ulceration is a global public health issue, with a prevalence of 13.0% in North America and 5.1% in Europe [7]. The incidence in Europe is around 2% per year [8]. Costs of care are double those seen in individuals without ulceration [9], with the total cost associated with treatment being as high as €10 billion per year in Europe [8]. The incidence of foot ulcers is 2.2% per annum in the UK, and an average of 6000 people with diabetes each year undergo amputation [10, 11]. The financial burden is significant in terms of both healthcare costs and long-term consequences. The annual UK National Health Service expenditure on diabetes foot-related care in 2010–2011 was estimated to be £580 million (US$748 million), representing around 0.6% of total expenditure [12]. Within 5 years (2014–2015), after accounting for inflation, estimates rose to between £972 million and £1.13 billion (US$1.29–1.50 billion), with two-thirds of these costs spent on care for foot ulceration in primary, community and outpatient settings [13]. Consequently, better precision in understanding the risk calculus in relation to the development of diabetic foot ulcers and the likelihood of death in such individuals will result in reduced health service costs and potential cost savings in the longer term.

Previous epidemiological studies have shown a strong association between socioeconomic disadvantage and the prevalence of diabetes [14, 15]. In the general population, mortality is greater in those from a more disadvantaged socioeconomic situation, principally as a result of cardiovascular disease [16]. In the presence of diabetes, however, mortality rates are significantly greater in individuals who live in relatively deprived areas [17]. Furthermore, depression has been found to be associated with a 2.3-fold increase in mortality vs no depression in individuals with diabetes [18]. Psychological factors other than coded diagnoses are difficult to quantify in epidemiological studies, but are clearly important.

The literature on the association between social deprivation and foot ulceration in individuals with diabetes is inconsistent. Two UK studies have shown no association between socioeconomic disadvantage and foot ulceration [19, 20]. Conversely, one Scottish study considered healthcare accessibility as well as socioeconomic disadvantage in the population and demonstrated evidence of increased foot ulceration in the more disadvantaged areas within Scotland [21]. The Eurodiale study has reported that low health-related quality of life is predictive of major amputation and mortality [22].

Few studies have looked at social disadvantage as an independent risk factor in diabetes foot ulceration and as a risk factor for mortality in those with a foot ulcer. The first aim of this study was to determine whether social deprivation is an independent predictor of foot ulceration in people with diabetes in a population with significantly varying levels of social disadvantage. Second, we tested the hypothesis that foot ulceration and level of social disadvantage independently predict all-cause mortality in individuals with type 2 diabetes.

## Methods

We examined pseudo-anonymised electronic health records from a retrospective cohort of all men and women aged 16–89 years attending 42 general practices in central and eastern Cheshire, UK. The area is a mixed urban and rural environment with a wide range of socioeconomic situations, from significantly disadvantaged urban areas to highly affluent suburbs. The total population of the geographical area studied was 475,000, and the prevalence of significant social disadvantage (based on multiple measures) was 23%.

Individuals were eligible for inclusion if they had a diagnosis of diabetes prior to cohort entry at 1 January 2004 to allow long-term follow-up and no prior history of foot ulceration. A data search was performed through the centralised data facility afforded by Egton Medical Information Systems (EMIS), a commercial organisation that provides health information for nearly all family practices in Cheshire. Permission for this study was sought from and granted by the local information governance and ethics committees. Informed consent was not required as all data were anonymised.

### Exposure

For the main search, we included all individuals using the relevant READ codes for type 1 and type 2 diabetes. READ codes are used in a hierarchical clinical coding system of more than 80,000 terms that is used in general practice across the UK [23]. The READ codes used in this study are available from the Clinical Codes repository (www.clinicalcodes.org) [24]. We examined the electronic health records of the included individuals to determine the presence of foot ulcers occurring after 1 January 2004 using the relevant READ codes.

### Outcomes

For all individuals, the outcomes were foot ulceration and all-cause mortality during the study period. Currently, the reporting of deaths within UK primary care is well established, and if an individual dies in secondary or tertiary care then a general practitioner (GP) must be notified of the death. The date of death was ascertained from GP records. We controlled for survival bias by following all participants from the same point in time: a landmark of 6 months post 1 January 2004. Follow-up was censored at death, the date an individual left the practice or the final data collection for the practice (30 June 2015), whichever occurred first.

### Variables

Data on potential confounders, including age on the date of inclusion, sex, diabetes type and duration, hypertension (defined as BP >140/90 mmHg on two or more readings, as per READ codes), diabetes treatment (oral medication, glucagon-like peptide 1 or insulin), metabolic control (through HbA_1c_) and smoking habits (unreported status, current/ex-smoker and non-smoker), were recorded, as was the prescribing of statins and antihypertensive agents.

The Townsend index of deprivation (categorised by quintiles) was determined in our study population [25]. This information is available in GP records linking the postcode of the individual with UK census data. The Townsend index was devised to provide a material measure of deprivation and socioeconomic disadvantage in a population, and derives from census variables taken originally from the 1991 UK census, with numerically higher values suggesting greater socioeconomic deprivation. The four variables that comprise the Townsend index are as follows: (1) unemployment as a percentage of those aged 16 years and over who are economically active; (2) non-car ownership as a percentage of all households; (3) non-home ownership as a percentage of all households; and (4) household overcrowding. All four variables are standardised using a *z* score and then summed to obtain a single value. Positive values of the Townsend index are associated with geographic areas with high deprivation, while indices with negative values relate to relative affluence. While the central and eastern Cheshire region is predominantly of white European ethnicity, records pertaining to ethnicity were only available for a small proportion (<4%) of the population and have therefore not been included.

### Assays

Blood glucose, lipid profile and serum creatinine were determined using automated clinical chemistry analysers. Samples assayed at Leighton Hospital used Ortho VITROS chemistry analysers (Ortho Clinical Diagnostics, High Wycombe, UK), and those assayed at Macclesfield District General Hospital used Beckman Coulter chemistry analysers (High Wycombe, UK). HbA_1c_ was determined using the using the Menarini Hb9210 semi-automated analyser (Menarini Diagnostics, Winnersh, UK). eGFR was calculated using the abbreviated modification of diet in renal disease equation [26]: eGFR (ml^−1^ min^−1^ 1.73 m^−2^) = 186 × (creatinine/88.4)^−1.154^ × (age)^−0.203^ × (0.742 if female) × (1.210 if black).

### Statistical analyses

Data were analysed using the statistical package Intercooled Stata version 13.1 (StataCorp, College Station, TX, USA). Data are expressed as mean (95% CI), mean (range), median (range) or number (percentage) where relevant. A linear regression analysis was used to determine the association between age and the presence of foot ulceration. We used the χ^2^ test or Fisher’s exact test to compare categorical variables, *t* tests or ANOVA for comparing continuous variables. Kaplan–Meier curves were used to compare survival probabilities for men and women with or without a history of foot ulceration. We investigated proportional hazards assumptions using tests and graphical diagnostics based on scaled Schoenfeld residuals. A test of the proportional hazards assumption was obtained by correlating the corresponding set of scaled Schoenfeld residuals with the Kaplan–Meier estimate of the survival distribution.

We fitted Poisson regressions from the occurrence (count) of an incident foot ulceration episode (per person) during follow-up, and report unadjusted and age-adjusted incidence rate ratios (and their 95% CI) from transformed coefficients using the Poisson command in Stata. An assessment of equidispersion was performed using post-estimation tests (data not shown), following Poisson regression for deviance goodness of fit and Pearson goodness of fit. We used logistic regression analyses to estimate the ORs and 95% CI for developing foot ulceration by quintile of Townsend index score.

We developed shared frailty multivariable survival Cox proportional hazard models, clustering for GP practice, to estimate HRs (and their 95% CI) for mortality in those with and without foot ulceration during the study period, adjusting for age (per year), sex, HbA_1c_ (per 10 mmol/mol), type of diabetes, smoking status, a history of cerebrovascular disease or myocardial infarction, hypertension, and a history of statin, aspirin, β-blocker or metformin prescription. We tested for interactions by adding terms for foot ulceration and Townsend index score (per quintile). Tests for statistically significant interactions used the Wald test (Stata command *testparm*); if the interaction term was not significant, this was excluded from the final model. We used the Stata command *estat* concordance to calculate the rank parameters Harrell’s C and Somers’ D as a measure of the ordinal predictive power of a model [27].

## Results

A total of 13,955 individuals (mean age 69.4 years) with diabetes were included in the analyses (Table 1). Of these, 1370 (9.8%) had type 1 diabetes and 12,585 (90.2%) were diagnosed with type 2 diabetes (Table 2). The incidence of foot ulceration increased with age (β = 4.3, *p* < 0.001), was more common in the elderly (>70 years old) and in individuals with type 2 compared with type 1 diabetes (8.6% [*n* = 1081] vs 4.8% [*n* = 66], χ^2^ = 23.3; *p* < 0.001). In the full cohort, stratified by sex, the occurrence of foot ulceration rate was similar (508/6011 [8.4%]) in women and men (639/7944, [8.0%]). However, amongst those who developed foot ulceration [*n* = 1147], a greater proportion were male (55.7% vs 44.3%; male versus female, *p* = 0.0001; Tables 1 and 2). At baseline, greater proportions of individuals with foot ulceration had hypertension, cerebrovascular disease, peripheral vascular disease and a previous myocardial infarction (all *p* < 0.01, Table 1). Mean systolic and diastolic blood pressure and levels of creatinine and LDL-cholesterol were greater in the presence of foot ulceration in individuals with type 2 diabetes (Table 2). No differences were observed in type 1 diabetes for these covariates.

**Table 1:** Baseline characteristics of all individuals by the presence or absence of foot ulceration

Variable	Total population		
	Foot ulcer^a^	No foot ulcer	*p* value^b^
	(*n*=1147)	(*n*=12,808)	
Age, years	74.2 (73.3, 74.2)	69.0 (68.8, 69.3)	<0.001
Sex, *n* (%)			
Male	639 (55.7)	7305 (57.0)	0.518
Female	508 (44.3)	5503 (43.0)	0.565
Age categories, *n* (%)			
<40 years	15 (1.3)	690 (5.4)	0.485
40–49 years	40 (3.5)	773 (6.0)	0.505
50–59 years	112 (9.8)	1495 (11.7)	0.542
60–69 years	194 (16.9)	2582 (20.2)	0.274
>70 years	786 (68.5)	7268 (56.7)	<0.001
Hypertension, *n* (%)	815 (71.0)	7704 (60.2)	<0.001
Cerebrovascular disease, *n* (%)	124 (10.8)	836 (6.5)	<0.001
Peripheral vascular disease, *n* (%)	168 (14.7)	535 (4.2)	<0.001
Previous myocardial infarction, *n* (%)	209 (18.2)	1483 (11.6)	<0.001
Congestive cardiac failure, *n* (%)	224 (19.5)	997 (7.8)	<0.001
Smoking status, *n* (%)			
Current/ex-smoker	375 (32.7)	4548 (35.5)	0.272
Non-smoker	427 (37.2)	3748 (29.3)	0.001
Unreported smoking status	345 (30.1)	4512 (35.2)	0.053
HbA_1c_, mmol/mol	60.7 (59.7, 61.8)	59.4 (59.1, 59.8)	0.024
HbA_1c_, %	7.7 (7.6, 7.8)	7.6 (7.6, 7.6)	0.024
Systolic BP, mmHg	143 (142, 147)	141 (140, 142)	<0.001
Diastolic BP, mmHg	78 (77, 79)	79 (78, 79)	0.01
Creatinine, μmol/l	100 (97, 102)	91 (90, 92)	<0.001
Total cholesterol, mmol/l	4.6 (4.5, 4.6)	4.7 (4.6, 4.7)	0.027
LDL-cholesterol, mmol/l	2.6 (2.5, 2.6)	2.7 (2.6, 2.7)	0.009
HDL-cholesterol, mmol/l	1.24 (1.21, 1.27)	1.27 (1.27, 1.28)	0.061
Statin treatment, *n* (%)	675 (58.9)	6847 (53.5)	<0.001
Townsend index score	−0.6 (−0.7, −0.4)	−1.2 (−1.2, −1.1)	<0.001

Data are the most recent measure within 1 year of study entry

Continuous data are presented as mean (95% CI)

^a^Foot ulceration identified during follow-up

^b^From test of proportions, χ^2^ tests (categorical variables) or ANOVA (continuous) for differences by the presence or absence of foot ulceration

**Table 2:** Baseline characteristics of all individuals by type of diabetes and foot ulceration status

Variable	Type 2 diabetes			Type 1 diabetes		
	Foot ulcer^a^	No foot ulcer	*p* value^b^	Foot ulcer^a^	No foot ulcer	*p* value^b^
	(*n*=1081)	(*n*=11,504)		(*n*=66)	(*n*=1304)	
Age, years	74.2 (73.5, 74.9)	71.7 (71.5, 72.0)	<0.001	59.33 (55.3, 63.4)	45.3 (44.4, 46.2)	<0.001
Sex, *n* (%)						
Male	594 (54.9)	6569 (57.1)	0.322	45 (68.2)	736 (56.4)	0.122
Female	487 (45.1)	4935 (42.9)	0.350	21 (31.8)	568 (43.6)	0.286
Age categories, *n* (%)						
<40 years	8 (0.7)	189 (1.6)	0.840	7 (10.6)	501 (38.4)	0.132
40–49 years	25 (2.3)	446 (3.9)	0.684	15 (22.7)	327 (25.1)	0.837
50–59 years	102 (9.4)	1260 (11.0)	0.618	10 (15.2)	235 (18.0)	0.817
60–69 years	182 (16.8)	2456 (21.4)	0.142	12 (18.2)	126 (9.7)	0.356
>70 years	764 (70.7)	7153 (62.2)	<0.001	22 (33.3)	115 (8.8)	0.002
Hypertension, *n* (%)	791 (73.2)	7478 (65.0)	<0.001	24 (36.4)	226 (17.3)	<0.001
Cerebrovascular disease, *n* (%)	118 (10.9)	828 (7.2)	<0.001	6 (9.1)	8 (0.6)	<0.001
Peripheral vascular disease, *n* (%)	152 (14.1)	502 (4.4)	<0.001	16 (24.2)	33 (2.5)	<0.001
Previous myocardial infarction, *n* (%)	203 (18.8)	1445 (12.6)	<0.001	6 (9.1)	38 (2.9)	0.005
Congestive cardiac failure, *n* (%)	220 (20.4)	979 (8.5)	<0.001	4 (6.1)	18 (1.4)	0.003
Smoking status, *n* (%)						
Current/ex-smoker	345 (31.9)	4062 (35.3)	0.204	30 (45.5)	486 (37.3)	<0.001
Non-smoker	427 (39.5)	3748 (32.6)	0.004	–	–	–
Unreported smoking status	309 (28.6)	3694 (32.1)	0.200	36 (54.5)	818 (62.7)	0.321
HbA_1c_, mmol/mol	60.1 (59.0, 61.1)	58.3 (57.9, 58.7)	0.003	72.4 (67.6, 77.2)	70.0 (68.9, 71.1)	0.341
HbA_1c_, %	7.6 (7.5, 7.7)	7.5 (7.4, 7.5)	0.003	8.8 (8.3, 9.2)	8.6 (8.5, 8.7)	0.34
Systolic BP, mmHg	144 (142, 145)	141 (141, 142)	0.002	136 (130, 142)	132 (130, 133)	0.175
Diastolic BP, mmHg	78 (77, 79)	79 (79, 79)	0.004	75 (71, 79)	75 (74, 76)	0.834
Creatinine, μmol/l	100 (96, 103)	91 (90, 92)	0.004	94 (78, 110)	89 (82, 91)	0.454
Total cholesterol, mmol/l	4.6 (4.5, 4.7)	4.6 (4.6, 4.7)	0.073	4.4 (4.0, 4.8)	4.8 (4.7, 4.9)	0.056
LDL-cholesterol, mmol/l	2.6 (2.5, 2.6)	2.6 (2.6, 2.7)	0.021	2.5 (2.1, 2.8)	2.8 (2.7, 2.9)	0.101
HDL-cholesterol, mmol/l	1.24 (1.21, 1.27)	1.26 (1.25, 1.26)	0.19	1.40 (1.18, 1.62)	1.52 (1.47, 1.57)	0.274
Statin treatment, *n* (%)	655 (60.6)	6605 (57.4)	0.043	20 (30.3)	242 (18.6)	0.018
Townsend index score	−0.6 (−0.8, −0.4)	−1.2 (−1.2, −1.1)	<0.001	−0.3 (−0.9, 0.4)	−1.3 (1.5, 1.2)	0.003

Data are the most recent measure within 1 year of study entry

Continuous data are presented as mean (95% CI)

^a^Foot ulceration identified during follow-up

^b^From test of proportions, χ^2^ tests (categorical variables) or ANOVA (continuous) for differences by the presence or absence of foot ulceration

### Social deprivation and foot ulceration rates

**Fig. 1:**
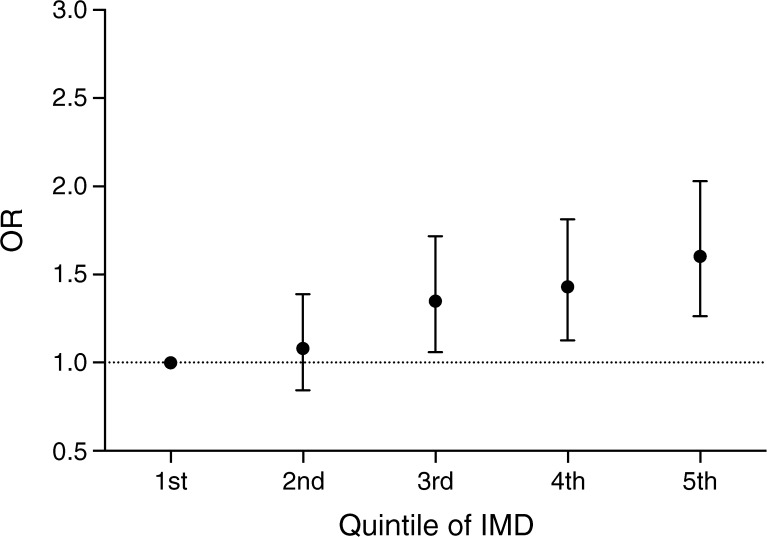
ORs (95% CI) for the likelihood of developing foot ulceration by quintile of index of multiple deprivation (IMD) in men and women with type 1 and type 2 diabetes

For those with foot ulceration, there were between one and 15 recorded episodes per person over the follow-up period, with an incidence rate of 0.9% per year. The unadjusted incidence rate ratio for a foot ulcer was 39% lower for individuals with type 1 vs type 2 diabetes (incidence rate ratio 0.61 [95% CI 0.47, 0.77], *p* < 0.001). Individuals in the fifth quintile of deprivation were 77% more likely to develop a foot ulcer over the follow-up period compared with those in the first quintile (OR 1.77 [95% CI 1.45, 2.14], *p* < 0.0001; Fig. 1).

### Foot ulceration rates and mortality

**Fig. 2:**
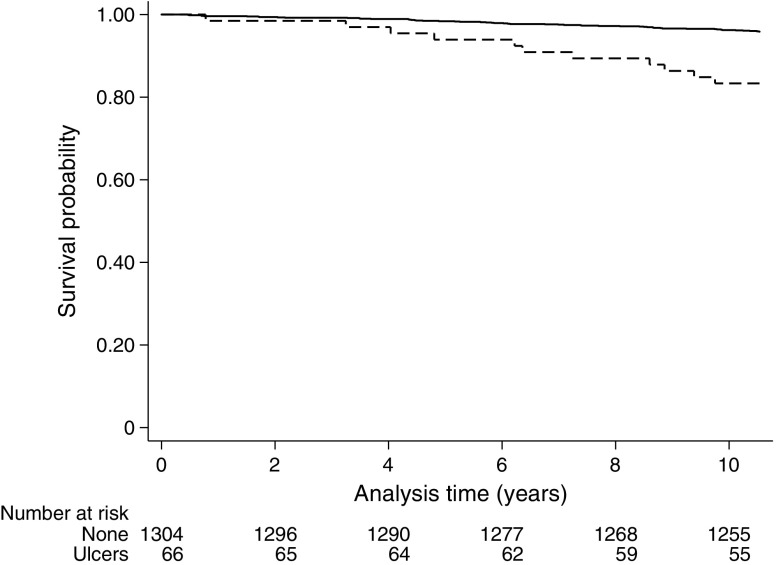
Kaplan–Meier curves for all-cause mortality for no foot ulceration (solid line) and foot ulceration (dashed line) in individuals with type 1 diabetes

**Fig. 3:**
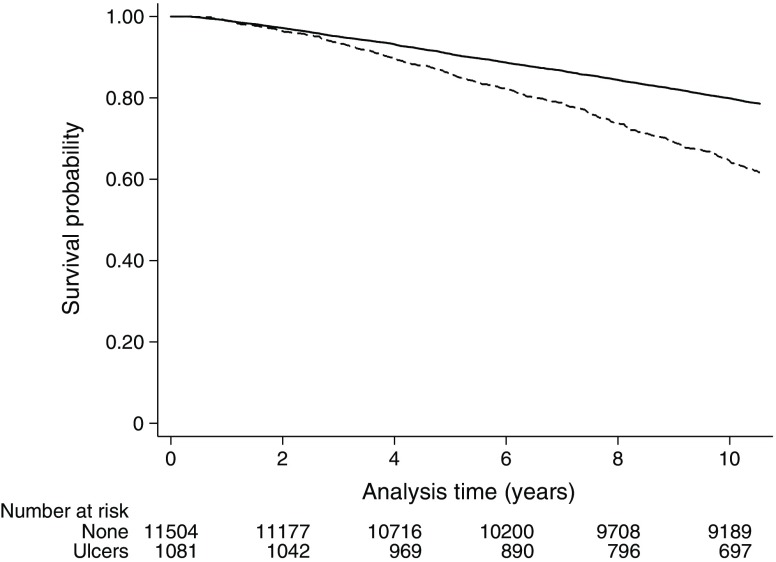
Kaplan–Meier curves for all-cause mortality for no foot ulceration (solid line) and foot ulceration (dashed line) in individuals with type 2 diabetes

Over a median follow-up period of 10.5 years there were 2946 (21.1%) deaths. Age- and sex-adjusted mortality rates in the group with foot ulcers were approximately twice those in the group without a foot ulcer (25.9% vs 14.0%; 41.6 vs 20.5 deaths per 1000 person-years, respectively). In the presence of type 2 and type 1 diabetes, the mortality rates for individuals with foot ulceration were 43.3 (95% CI 39.3, 47.7) and 16.9 (95% CI 9.3, 30.6) per 1000 person-years, respectively. Foot ulceration was associated with an increased risk of mortality in individuals with type 1 diabetes in unadjusted shared frailty Cox proportional hazard models clustered by practice (HR 4.45 [95% CI 2.29–8.64]; Fig. 2), but not after adjustment for age and sex (HR 1.55 [95% CI 0.79, 3.07], *p* = 0.20). For individuals with type 2 diabetes, there was an almost doubling of the risk of mortality in unadjusted shared frailty Cox regression analyses clustered by practice (HR 1.93 [95% CI 1.74–2.15], *p* < 0.0001; Fig. 3), with some attenuation of the size of the effect after age- and sex-adjustment (HR 1.71 [95% CI 1.54, 1.90], *p* < 0.001).

In similar age- and sex-adjusted models including only those with no prior/current history of foot ulceration, increased deprivation (per quintile) was associated with increased mortality in those with type 2 diabetes (HR 1.13 [95% CI 1.09, 1.16], *p* < 0.0001), but not in those with type 1 diabetes (HR 1.05 [95% CI 0.86, 1.27], *p* = 0.063; ESM Table 1).

### Social deprivation and death in individuals with foot ulcer

**Fig. 4:**
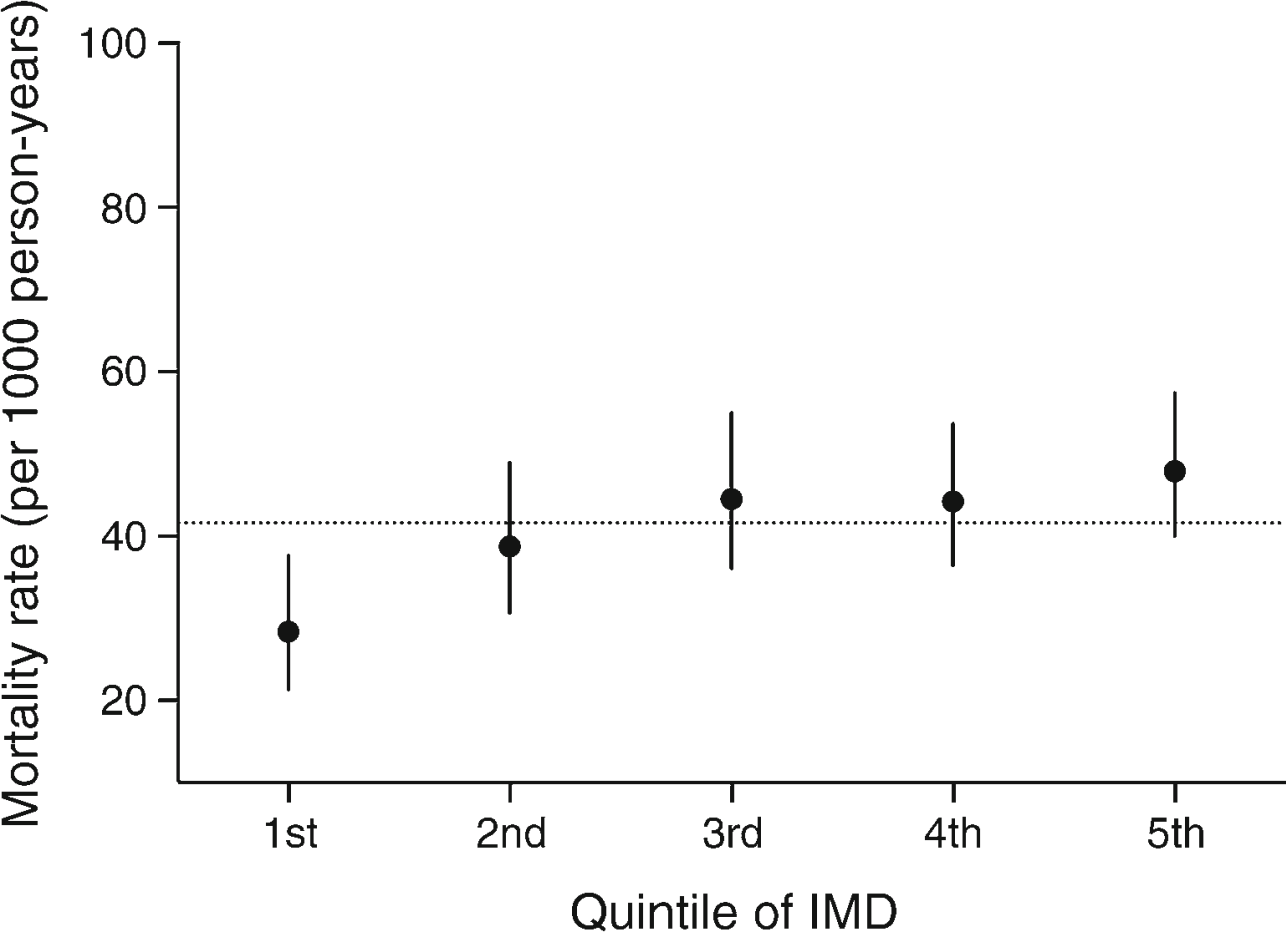
All-cause mortality rates (95% CI) for foot ulceration by quintile of index of multiple deprivation (IMD) in men and women with type 1 and type 2 diabetes. The horizontal dotted line represents the mean mortality rates (41.6 [95% CI 37.8, 45.8]) for men and women with foot ulceration

Death rates increased per quintile of deprivation in individuals with diabetes and foot ulceration (absolute difference between the first and fifth quintile 19.54 deaths per 1000 person-years; Fig. 4). Risk of death increased by 14% per quintile of deprivation in a univariable analysis (HR 1.14 [95% CI 1.10, 1.17%]) for all individuals. For individuals with type 2 diabetes, the risk of mortality in those who developed foot ulceration increased by 11% per quintile of deprivation (HR 1.11% [95% CI 1.08%, 1.14%], *p* < 0.0001). The association of deprivation with mortality in type 1 diabetes was not significant (HR 1.14 [95% CI 0.96, 1.36%], *p* = 0.136).

**Fig. 5:**
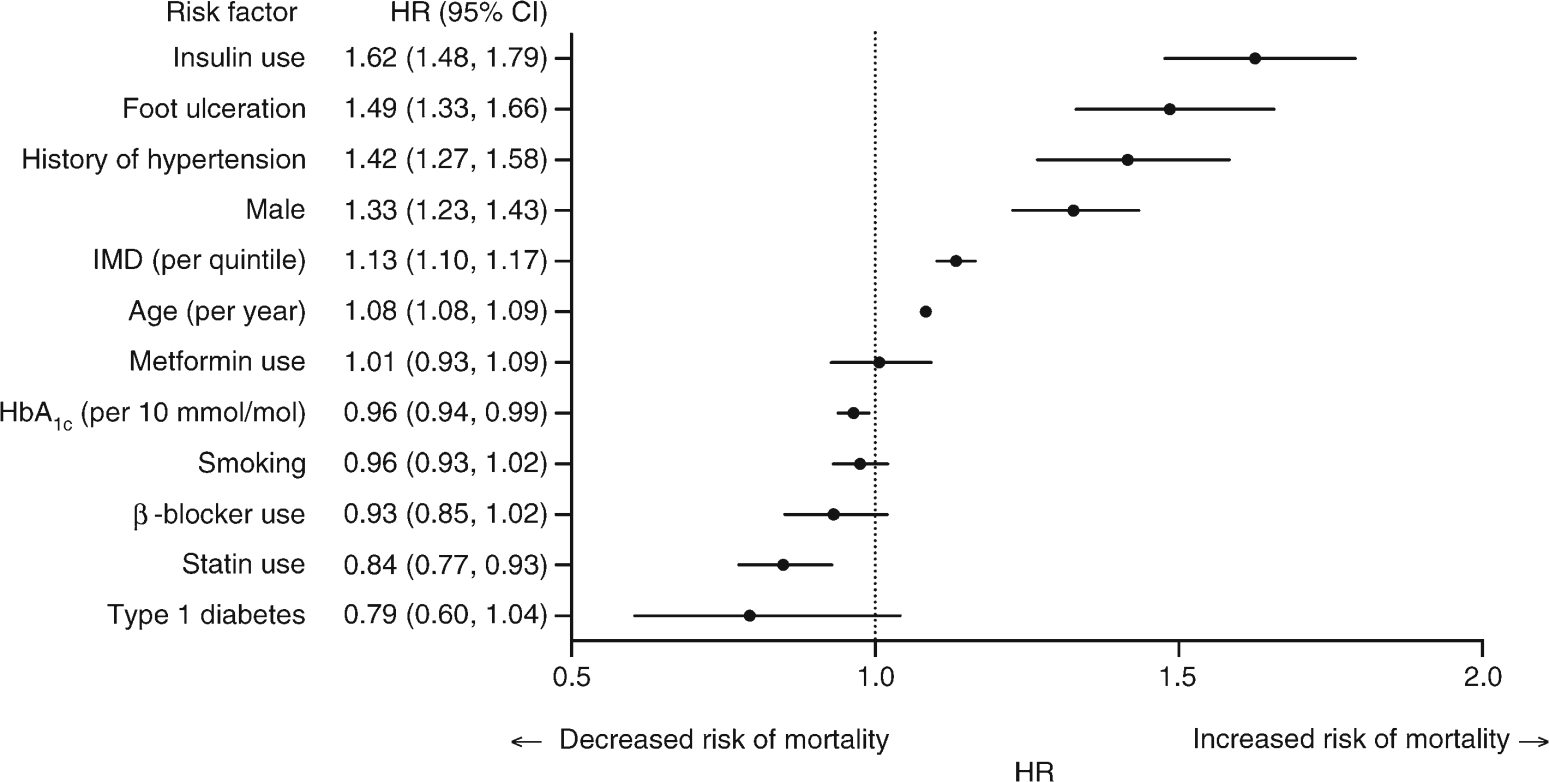
Forest plot of multivariable adjusted HRs of all-cause mortality associated with the presence of foot ulceration in men and women with type 2 diabetes. Cox regression models for risk of all-cause mortality were stratified by GP practice and adjusted for age, sex, diabetes type, smoking status, history of hypertension, β-blocker use, HbA_1c_ level, Townsend index of multiple deprivation (IMD) score, metformin use, insulin use and statin use. Harrell’s C concordance statistic = 0.73 for model

In shared frailty, multivariable Cox regression analyses clustered by practice, foot ulceration (HR 1.48 [95% CI 1.33, 1.66], *p* < 0.0001) independently predicted long-term mortality after adjustment for age, sex, diabetes type, Townsend index score (HR 1.13 [95% CI 1.10, 1.17], per quintile), smoking status, hypertension, statin treatment, β-blocker treatment, metformin use, HbA_1c_ level (per 10 mmol/mol) and insulin use (Fig. 5). There was no interaction between foot ulceration and the Townsend index score for deprivation (χ^2^ = 1.49; *p* for interaction = 0.475).

## Discussion

In this retrospective cohort followed for 10.5 years, individuals with diabetes-related foot ulceration had a higher risk of all-cause mortality than those without a history of foot ulceration. Specifically, after multivariable adjustment, a 48% increased risk of mortality was observed in those who developed a foot ulcer. We also found that social deprivation is an independent predictor of mortality, with the risk of death increasing by 13% per quintile of deprivation (HR 1.13 [95% CI 1.10, 1.17]) independent of baseline age, sex, diabetes type, smoking status, hypertension, statin, β-blocker or metformin use, HbA_1c_ level and insulin use. Individuals in the highest quintile of deprivation were 77% more likely to develop a foot ulcer compared with those in the lowest quintile.

Our finding demonstrate similar rates of foot ulcer incidence by sex (8.4% vs 8.0%, females vs males). The lower rates of foot ulceration seen here compared with previous studies may relate to the emphasis placed on foot screening and ulceration-preventative measures among individuals with diabetes in the Cheshire and High Peak Derbyshire areas of the UK over the duration of this study. Overall, our findings from real-world electronic medical records are comparable with those of previous epidemiological reports and add to the current body of evidence. The increased mortality reported here is consistent with previous reports [28–30], including a meta-analysis of 17,830 individuals with diabetes and with or without foot ulceration [31]. This included six prospective [32–37] and two retrospective cohorts [38, 39].

In the context of social disadvantage, Leese et al, in a Scottish study, showed a link between social disadvantage and diabetic foot disease [21], with the most deprived quintile having a 1.7-fold increased risk of developing a foot ulcer. Access to a GP or hospital clinic was not associated with foot ulceration or amputation. We have previously shown that socially disadvantaged individuals have a predisposition to developing painful peripheral neuropathy, an important determinant of foot ulceration in both type 1 and type 2 diabetes [40]. Regarding the increased death rate in individuals with an established foot ulcer, the dominant prognostic factor may be the effect of social disadvantage [41, 42]. Other factors include a greater intake of foods with a high glycaemic index and concomitant poorer glycaemic control [43].

Among individuals with diabetes, a previous study has reported that both minor and major depression are strongly associated with increased mortality [18]. Specifically, even after adjustment for age, sex, race/ethnicity and educational attainment, compared with the non-depressed group, minor and major depression were associated with a 1.67- and 2.30-fold increase in mortality, respectively. Depression itself was associated with greater socioeconomic disadvantage and lower educational attainment.

The current study has several strengths and weaknesses. It was a longitudinal study of all individuals attending GP practices in Cheshire, UK, with a median follow-up of 10.5 years. As a far as possible, GPs and practice nurses record any events and changes in medication. The area has a stable population, with little migration out of the area. One weakness is that, akin to all studies using primary-care data, our findings are subject to variability in the data entry of different GP practices. In relation to this, a record of more than one episode of foot ulceration could not be ascribed in all cases as true multiple episodes of new foot ulcers. Second, we did not have the actual anatomical location of the foot ulcers. There is potential for under-reporting of foot ulceration. However, the area of the UK included in this study has a highly proactive podiatry service, led by one of the co-authors (GD), that engages closely with GP practices in the diagnosis and treatment of diabetes-related foot problems. Furthermore, diabetes management has changed a lot since 2004, particularly with respect to more aggressive BP and lipid management, with recent studies showing that rates of cardiovascular death have declined. This issue affects prospective studies in this area. Another weakness is that there was no specific information in the GP-coded records on psychological symptoms (including depression), educational attainment and occupation, all of which could potentially affect foot ulceration rates and impact mortality.

Foot problems in diabetes continue to challenge the clinicians who care for these individuals. Not only are foot ulcers associated with morbidity and disability, but they also lead to significant impairment of quality of life [44]. It is known that poor health-related quality of life is a predictive factor for major amputation and mortality [22]. We found increased mortality in individuals with a history of foot ulceration, and this risk increased per quintile of deprivation. This key finding highlights the importance of targeting resources to less socioeconomically advantaged individuals with diabetes in any community, particularly when they have a foot ulcer.

A proactive multidisciplinary approach is warranted to manage foot problems in these individuals. Such an approach should involve actively targeting cardiovascular risk factors at the GP practice level using case identification. This may reduce the incidence of foot ulceration and, subsequently, the associated increased mortality. Recognising and reducing the risk of death from associated comorbid conditions can save not only the limb but also, ultimately, a person’s life. The educational attainment of the individual must be considered for them to appreciate the significance of their own diabetic foot ulceration. Data pertaining to educational attainment were not available for our analysis.

In summary, this study confirms the high mortality of individuals with diabetes-related foot ulcers, in addition to the well established associated substantial morbidity and disability. Socioeconomic disadvantage is an independent predictor of mortality in individuals with diabetes with a foot ulcer and of the occurrence of foot ulceration in people with diabetes. This highlights the importance of taking socioeconomic disadvantage into account when planning future diabetes services.

## Electronic supplementary material

ESM (PDF 133 kb)

## Abbreviation

GP: General practitioner

## References

[1] Boulton AJ, Armstrong DG, Albert SF (2008). Comprehensive foot examination and risk assessment. A report of the Task Force of the Foot Care Interest Group of the American Diabetes Association, with endorsement by the American Association of Clinical Endocrinologists. Phys Ther.

[2] Diabetes UK (2010). Key statistics on diabetes.

[3] Boulton AJ, Vileikyte L, Ragnarson-Tennvall G, Apelqvist J (2005). The global burden of diabetic foot disease. Lancet.

[4] Rao Kondapally Seshasai S, Kaptoge S, Thompson A (2011). Diabetes mellitus, fasting glucose, and risk of cause-specific death. N Engl J Med.

[5] Ghanassia E, Villon L, Thuan Dit Dieudonne JF, Boegner C, Avignon A, Sultan A (2008). Long-term outcome and disability of diabetic patients hospitalized for diabetic foot ulcers: a 6.5-year follow-up study. Diabetes Care.

[6] Boulton AJ, Kirsner RS, Vileikyte L (2004). Clinical practice. Neuropathic diabetic foot ulcers. N Engl J Med.

[7] Zhang P, Lu J, Jing Y, Tang S, Zhu D, Bi Y (2017). Global epidemiology of diabetic foot ulceration: a systematic review and meta-analysis. Ann Med.

[8] Prompers L, Huijberts M, Schaper N (2008). Resource utilisation and costs associated with the treatment of diabetic foot ulcers. Prospective data from the Eurodiale Study. Diabetologia.

[9] Rice JB, Desai U, Cummings AK, Birnbaum HG, Skornicki M, Parsons NB (2014). Burden of diabetic foot ulcers for medicare and private insurers. Diabetes Care.

[10] NHS Digital (2007) Hospital episode statistics. Available from www.hscic.gov.uk. Accessed 12 April 2016

[11] Abbott CA, Carrington AL, Ashe H (2002). The North-West Diabetes Foot Care Study: incidence of, and risk factors for, new diabetic foot ulceration in a community-based patient cohort. Diabet Med.

[12] Kerr M, Rayman G, Jeffcoate WJ (2014). Cost of diabetic foot disease to the National Health Service in England. Diabet Med.

[13] Kerr M (2017) Improving footcare for people with diabetes and saving money: an economic study in England. Available from https://diabetes-resources-production.s3-eu-west-1.amazonaws.com/diabetes-storage/migration/pdf/Improving%2520footcare%2520economic%2520study%2520%28January%25202017%29.pdf. Accessed 16 October 2017

[14] Connolly V, Unwin N, Sherriff P, Bilous R, Kelly W (2000). Diabetes prevalence and socioeconomic status: a population based study showing increased prevalence of type 2 diabetes mellitus in deprived areas. J Epidemiol Community Health.

[15] Baumer JH, Hunt LP, Shield JP (1998). Social disadvantage, family composition, and diabetes mellitus: prevalence and outcome. Arch Dis Child.

[16] Chaturvedi N, Jarrett J, Shipley MJ, Fuller JH (1998). Socioeconomic gradient in morbidity and mortality in people with diabetes: cohort study findings from the Whitehall Study and the WHO Multinational Study of Vascular Disease in Diabetes. BMJ.

[17] Weng C, Coppini DV, Sonksen PH (2000). Geographic and social factors are related to increased morbidity and mortality rates in diabetic patients. Diabet Med.

[18] Katon WJ, Rutter C, Simon G (2005). The association of comorbid depression with mortality in patients with type 2 diabetes. Diabetes Care.

[19] Ince P, Kendrick D, Game F, Jeffcoate W (2007). The association between baseline characteristics and the outcome of foot lesions in a UK population with diabetes. Diabet Med.

[20] Vaidya B, Roper NA, Connolly VM, Kelly WF (2003). Socio-economic deprivation and diabetic foot ulcers: no strong association. Diabet Med.

[21] Leese GP, Feng Z, Leese RM, Dibben C, Emslie-Smith A (2013). Impact of health-care accessibility and social deprivation on diabetes related foot disease. Diabet Med.

[22] Siersma V, Thorsen H, Holstein PE (2014). Health-related quality of life predicts major amputation and death, but not healing, in people with diabetes presenting with foot ulcers: the Eurodiale study. Diabetes Care.

[23] Chisholm J (1990). The Read clinical classification. BMJ.

[24] Springate DA, Kontopantelis E, Ashcroft DM (2014). ClinicalCodes: an online clinical codes repository to improve the validity and reproducibility of research using electronic medical records. PLoS One.

[25] Townsend P, Phillimore P, Beattie A (1988). Health and Deprivation: Inequality and the North.

[26] Levey AS, Bosch JP, Lewis JB, Greene T, Rogers N, Roth D (1999). A more accurate method to estimate glomerular filtration rate from serum creatinine: a new prediction equation. Modification of Diet in Renal Disease Study Group. Ann Intern Med.

[27] Newson RB (2010). Comparing the predictive powers of survival models using Harrell’s C or Somers’ D. Stata J.

[28] Jiang Y, Wang X, Xia L (2015). A cohort study of diabetic patients and diabetic foot ulceration patients in China. Wound Repair Regen.

[29] Martins-Mendes D, Monteiro-Soares M, Boyko EJ (2014). The independent contribution of diabetic foot ulcer on lower extremity amputation and mortality risk. J Diabetes Complicat.

[30] Nirantharakumar K, Saeed M, Wilson I, Marshall T, Coleman JJ (2013). In-hospital mortality and length of stay in patients with diabetes having foot disease. J Diabetes Complicat.

[31] Brownrigg JR, Davey J, Holt PJ (2012). The association of ulceration of the foot with cardiovascular and all-cause mortality in patients with diabetes: a meta-analysis. Diabetologia.

[32] Boyko EJ, Ahroni JH, Smith DG, Davignon D (1996). Increased mortality associated with diabetic foot ulcer. Diabet Med.

[33] Pham H, Armstrong DG, Harvey C, Harkless LB, Giurini JM, Veves A (2000). Screening techniques to identify people at high risk for diabetic foot ulceration: a prospective multicenter trial. Diabetes Care.

[34] Davis WA, Norman PE, Bruce DG, Davis TM (2006). Predictors, consequences and costs of diabetes-related lower extremity amputation complicating type 2 diabetes: the Fremantle Diabetes Study. Diabetologia.

[35] Pinto A, Tuttolomondo A, Di Raimondo D (2008). Cardiovascular risk profile and morbidity in subjects affected by type 2 diabetes mellitus with and without diabetic foot. Metabolism.

[36] Junrungsee S, Kosachunhanun N, Wongthanee A, Rerkasem K (2011). History of foot ulcers increases mortality among patients with diabetes in Northern Thailand. Diabet Med.

[37] Iversen MM, Tell GS, Riise T (2009). History of foot ulcer increases mortality among individuals with diabetes: ten-year follow-up of the Nord-Trondelag Health Study, Norway. Diabetes Care.

[38] Ramsey SD, Newton K, Blough D (1999). Incidence, outcomes, and cost of foot ulcers in patients with diabetes. Diabetes Care.

[39] Sohn MW, Lee TA, Stuck RM, Frykberg RG, Budiman-Mak E (2009). Mortality risk of Charcot arthropathy compared with that of diabetic foot ulcer and diabetes alone. Diabetes Care.

[40] Anderson SG, Narayanan RP, Malipatil NS, Roberts H, Dunn G, Heald AH (2015). Socioeconomic deprivation independently predicts painful diabetic neuropathy in type 2 diabetes. Exp Clin Endocrinol Diabetes.

[41] Chaturvedi N, Stevens LK, Fuller JH, Lee ET, Lu M (2001). Risk factors, ethnic differences and mortality associated with lower-extremity gangrene and amputation in diabetes. The WHO Multinational Study of Vascular Disease in Diabetes. Diabetologia.

[42] Leymarie F, Richard JL, Malgrange D (2005). Factors associated with diabetic patients at high risk for foot ulceration. Diabete Metab.

[43] Konttinen H, Sarlio-Lahteenkorva S, Silventoinen K, Mannisto S, Haukkala A (2013). Socio-economic disparities in the consumption of vegetables, fruit and energy-dense foods: the role of motive priorities. Public Health Nutr.

[44] Ragnarson Tennvall G, Apelqvist J (2000). Health-related quality of life in patients with diabetes mellitus and foot ulcers. J Diabetes Complicat.

