# Supplementary material for: Social deprivation modifies the association between incident foot ulceration and mortality in type 1 and type 2 diabetes: a longitudinal study of a primary-care cohort
Source: Diabetologia. 2017 Dec 21;61(4):959–67. doi: 10.1007/s00125-017-4522-x (PMC6448990; doi:10.1007/s00125-017-4522-x)
Supplement: Supplementary file 1 — (PDF 133 kb) [file 125_2017_4522_MOESM1_ESM.pdf]

Social deprivation modifies the association between incident foot ulceration and mortality in type 1 and type 2 diabetes: a longitudinal study of a primary-care cohort

Simon G. Anderson[1], Haika Shoo[2], Sushant Saluja[1], Christian D. Anderson[3], Adnan Khan[4], Mark Livingston[5], Edward B. Jude[6], Mark Lunt[7], George Dunn[8] and Adrian H. Heald[9,10]

1. Division of Cardiovascular Sciences, Faculty of Biology, Medicine, and Health, University of Manchester, Manchester, UK

2. Diabetes and Endocrine Department, East Cheshire NHS Trust, Macclesfield, UK

3. School of Medicine, University of Liverpool, Liverpool, UK

4. Department of Endocrinology and Diabetes, Leighton Hospital, Crewe, UK

5. Department of Blood Sciences, Walsall Manor Hospital, Walsall, UK

6. Department of Diabetes and Endocrinology, Tameside Hospital NHS Foundation Trust, Ashton-under-Lyne, UK

7. Arthritis Research UK Centre for Epidemiology, Centre for Musculoskeletal Research, School of Biological Sciences and Manchester Academic Health Science Centre, University of Manchester, UK

8. Department of Podiatry, East Cheshire NHS Trust, Macclesfield, UK

9. Salford Royal NHS Foundation Trust, Diabetes and Endocrinology, Stott Lane, Salford, UK

10. School of Medical Sciences, Faculty of Biology, Medicine, and Health, and Manchester Academic Health Science Centre (MAHSC), The University of Manchester, 46 Grafton Street, Manchester, M13 9NT, UK

Corresponding author:

Dr. A. H. Heald,

School of Medical Sciences, Faculty of Biology, Medicine, and Health, and Manchester Academic Health Science Centre (MAHSC), The University of Manchester, 46 Grafton Street, Manchester, M13 9NT

email: adrian.heald@manchester.ac.uk

ESM Table 1.

Table showing hazard ratios for mortality from age-sex adjusted models for individuals with **no** prior/current history of foot ulceration for Type 2 (model A) and Type 1 diabetes (model B)

	Hazard Ratios	95% Conf. Interval		P value
**Type 2 Diabetes (N = 11,484)**				
First quintile	1.00	-	-	-
Second quintile	1.03	0.90	1.18	0.64
Third quintile	1.13	0.99	1.29	0.07
Fourth quintile	1.46	1.29	1.65	0.00
Fifth quintile	1.52	1.34	1.73	0.00
**Type 1 Diabetes (N = 1293)**				
First quintile	1.00	-	-	-
Second quintile	0.56	0.23	1.39	0.21
Third quintile	0.86	0.39	1.91	0.72
Fourth quintile	1.28	0.61	2.70	0.51
Fifth quintile	0.86	0.36	2.07	0.74
